# Pushing the Limits of Surface-Enhanced Raman Spectroscopy (SERS) with Deep Learning: Identification of Multiple Species with Closely Related Molecular Structures

**DOI:** 10.1177/00037028221077119

**Published:** 2022-03-26

**Authors:** Alexis Lebrun, Hubert Fortin, Nicolas Fontaine, Daniel Fillion, Olivier Barbier, Denis Boudreau

**Affiliations:** 1 Departement of Chemistry, 4440 Université Laval, Québec, Canada; 2 Center for Optics, Photonics and Lasers (COPL), Université Laval, Québec, Canada; 3 Laboratoire de Pharmacologie Moléculaire, Axe Endocrinologie-Néphrologie, Centre de recherche du CHU de Québec, 4440 Université Laval, Quebec, Canada

**Keywords:** Surface-enhanced Raman spectroscopy, SERS, deep learning, spectral classification, convolutional neural network, CNN

## Abstract

Raman spectroscopy is a non-destructive and label-free molecular identification technique capable of producing highly specific spectra with various bands correlated to molecular structure. Moreover, the enhanced detection sensitivity offered by surface-enhanced Raman spectroscopy (SERS) allows analyzing mixtures of related chemical species in a relatively short measurement time. Combining SERS with deep learning algorithms allows in some cases to increase detection and classification capabilities even further. The present study evaluates the potential of applying deep learning algorithms to SERS spectroscopy to differentiate and classify different species of bile acids, a large family of molecules with low Raman cross sections and molecular structures that often differ by a single hydroxyl group. Moreover, the study of these molecules is of interest for the medical community since they have distinct pathological roles and are currently viewed as potential markers of gut microbiome imbalances. A convolutional neural network model was developed and used to classify SERS spectra from five bile acid species. The model succeeded in identifying the five analytes despite very similar molecular structures and was found to be reliable even at low analyte concentrations.

## Graphical abstract

**Graphical Abstract:**
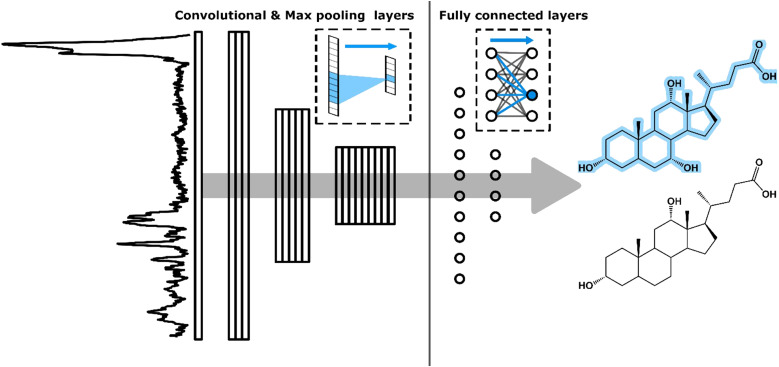

## Introduction

Surface enhanced Raman spectroscopy (SERS) is a powerful vibrational analysis technique able to detect and identify molecular species with high sensitivity. By exploiting the localized surface plasmon resonance offered by noble metal nanoparticles, SERS can enhance the intrinsically low sensitivity of Raman spectroscopy by up to nine orders of magnitude,^1–3^ allowing the detection of molecules at lower concentrations (or with lower laser power and shorter integration times) in various fields of research, including the detection of various molecular species in biological samples.^4,5^

Despite the widespread use of SERS, a large number of molecular targets, including biomarkers of interest to the medical community, remain unexplored. Among these, bile acids (BAs) are a large family of molecules that includes species with closely related structures and distinct physiological properties. They are synthesized exclusively in the liver from cholesterol, stored in the gallbladder, and released into the gut during meals. Cholesterol conversion leads to the formation of the primary BAs synthesized in the human liver, namely, cholic acid and chenodeoxycholic acid.^6^ These acids are then conjugated to taurine (in rodents) or glycine (in humans) to serve as natural detergents by facilitating the intestinal absorption of fatty acids and fat-soluble vitamins contained in foods.^6,7^ Evaluating their intestinal metabolism is therefore extremely relevant to estimate how several stimuli (such as diets, for example) will affect such physiological functions. By contrast, the secondary acid, lithocholic acid, is a potent activator of the intestinal vitamin D receptor and, by doing so, acts as an important intestinal immunomodulator, while deoxycholic acid is a natural antibiotic that contributes to maintain the intestinal microbiome diversity.^6^ In the same vein, by mirroring the activity level of the bacterial bile salt hydrolase, the measurement of taurochenodeoxycholic and glycochenodeoxycholic acids in the intestine provides essential information on microbiota health.^8–11^ Thus, intestinal dysbiosis (i.e., alteration of microbiota homeostasis) often leads to significant changes in BA concentration profiles, which in turn contributes to the development of important intestine pathologies such as intestinal bowel diseases (IBD) and colorectal cancers.^8^

Most studies on bile acid profiling published to date have used mass spectrometry combined with liquid chromatography (LC-MS).^12,13^ Although LC-MS provides excellent sensitivity and selectivity, it requires considerable expertise and expensive instrumentation. Moreover, whereas these approaches are usable only for fecal profiling, most of the BAs secreted in the intestine are reabsorbed in the ileum and returned to the liver through the portal circulation.^6^ Thus, profiling bile acids in feces unavoidably provides inaccurate estimations of their intestinal levels. Therefore, it is urgently needed to implement novel BA measurement technologies allowing for in situ profiling of intestinal bile acids. To this end, SERS is an interesting candidate for BA profiling as it is relatively fast and requires simpler and less expensive simple instrumentation.

Classifying distinct spectral signatures from a mixture of BAs is a difficult challenge as some species only differ by the presence or absence of a single hydroxyl group and present exceedingly similar Raman spectra (Fig. 4a). Various statistical methods, for example, principal component analysis (PCA) and partial least squares regression, can be used to assist spectral analysis.^14,15^ Standard machine learning models can be used as well, but an increasing number of studies are now using deep neural networks since they outperform standard machine learning models and previous statistical methods in most cases.^16–19^ The application of SERS spectroscopy in conjunction with a deep learning model for the difficult task of identifying different bile acids is therefore an excellent opportunity to validate and develop the potential of this combination of techniques.

In the work presented here, a convolutional neural network (CNN) model was developed to classify bile acids from their SERS spectra. This model was applied to the classification of SERS spectra of five different bile acids, namely, cholic (CA), glycochenodeoxycholic (GCDCA), taurochenodeoxycholic (TCDCA), deoxycholic (DCA), and lithocholic (LCA) acids. These compounds were selected as markers for unconjugated (CA, DCA, and LCA), conjugated (GCDCA and TCDCA), and primary (CA, GCDCA, and TCDCA) or secondary (DCA and LCA) acids of human (CA, GCDCA, DCA, and LCA) or murine (CA, TCDCA, DCA, and LCA) origin.^6^ The complete development cycle, from the synthesis of metal nanoparticles and the preparation of SERS substrates to the training of the model, validation, and testing with BA mixtures at various concentrations is presented.

## Material and Methods

All reagents were purchased from Sigma Aldrich unless specified otherwise. GCDCA and TCDCA came from ArchPharm and Milipopo, respectively. Anhydrous ethanol (99.9%) and Milli-Q water with a resistivity of 18.2 MΩ were used for all experiments. Standard silica microscope coverslips (25 × 30 mm) were used as substrates for the immobilization of gold nanostars (AuNSt).

### Synthesis of Gold Nanostars

Gold nanostars (AuNSt) were synthesized following a slightly modified one-pot seedless protocol,^20,21^ using gold (III) chloride (HAuCl_4_, 99% purity), with silver nitrate (AgNO_3_, 99.9999% purity) as shaping agent, and ascorbic acid as reducing agent. Briefly, 1.44 mL of 10 mM HAuCl_4_ aqueous solution was added to a 50 mL polypropylene tube containing 40 mL of water and vortexed for 10 s .80 µL of 10 mM aqueous AgNO_3_ was then mixed with the solution and vortexed for another 10 s. Finally, 240 µL of 100 mM ascorbic acid was added to the mixture followed by 20 s of vortex (the solution should change from translucent yellow to greenish blue). The synthesized AuNSt were centrifuged at 2500 RCF for 25 min, resuspended in 40 mL of water, and stored at 4 °C. Optical extinction spectra of the synthesized AuNSt were obtained using a Cary-5000 ultraviolet–visible (UV–Vis) spectrometer.

### Preparation of Surface-Enhanced Raman Spectroscopy Substrates

The SERS substrates were fabricated using a silanization process with (3-aminopropyl) triethoxysilane (APTES) to immobilize AuNSt on commercial microscope coverslips.^22,23^ Coverslips were pretreated in a piranha solution (3:1 H_2_SO_4_:H_2_O_2_) for 30 min to remove any organic impurities and then soaked for 15 min in a 1:1:1 H_2_O:NH_4_OH:H_2_O_2_ solution to increase the number of hydroxyl groups on the surface. The coverslips were rinsed thoroughly with water between these two steps and with water and ethanol before the silanization process. Silanization of the pretreated coverslip was carried out for 2 h in a 1% ethanolic APTES solution. Upon completion of the silanization, the coverslips were rinsed thoroughly with ethanol and dried for 1 h at 120 °C in an oven. A meniscus evaporation-assisted deposition process adapted from literature was used to improve AuNSt deposition.^24^ Briefly, a pair of glass coverslips was assembled into a thin chamber using a parafilm gasket (Fig. S1, Supplemental Material), the chamber was filled with concentrated AuNSt solution (1.5 mL of the initial AuNSt solution centrifuged at 2000 RCF for 25 min and resuspended in 20 µL of water) and dried under vacuum for 2 h. This process was repeated three times to obtain a dense, uniform, and reproducible AuNSt-coated coverslip.

These SERS substrates were functionalized with an anti-fouling and BA-selective layer composed of a mixture of two thiol capping agents, that is, 1-octanethiol (OCT) and 2-(dimethylamino)ethanethiol (DMAET). Inspired by naturally occurring intestinal ion-exchange resins and bile acid sequestrants,^25^ this mixture has high affinity for hydrophobic and anionic BAs, as it presents both positive charges (DMAET, protonated amines) and hydrophobic properties (OCT, long carbon chains) (Fig. 4c). The substrates were immersed overnight (18 h) in an ethanolic solution of OCT (0.05 mM) and DMAET (0.05 mM). They were then rinsed three times with ethanol, dried with a nitrogen stream and stored in a desiccator in the dark. SEM images of the substrates were measured on a Quanta-3D-FEG (FEI Inc.) and analyzed with the ImageJ software.

### Raman Instrumentation

**Figure 1.**
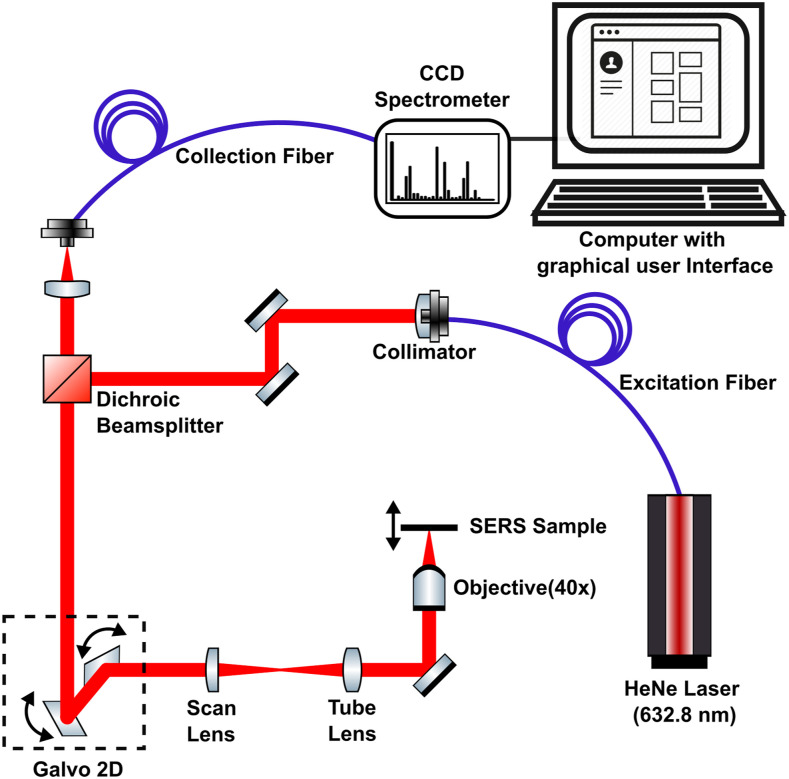
Diagram of the laboratory-made confocal Raman microscope used in this work.

A laboratory-made confocal Raman microscope was used for SERS measurements and is presented in Fig. 1. The excitation light source consisted of a 10 mW He–Ne laser (632.8 nm) injected into a single-mode optical fiber. An Olympus NiFluorite objective (M = 40 X, NA = 0.75) was used to excite the sample with an incident power of (2.8 ± 0.3) mW measured at the sample location. A galvanometer-based laser scanning apparatus allowed the interrogation of large areas more rapidly than a sample-scanning setup. A compact spectrometer (Fergie, Princeton Instruments) featuring a 1180 lines/mm grating blazed at 750 nm (spectral resolution ≅ 0.22 nm) and a near-infrared-enhanced charge-coupled device (CCD) detector cooled at −55 °C was connected to the microscope using a multimode fiber (OZ Optics) that also served as the microscope pinhole (φ = 50 µm). CCD images were acquired with a 5 s integration time and binned into 1 × 1024 spectra. The spectral range covered by the spectrometer in this configuration was 650–780 nm (416–3100 cm^−1^).

### Surface-Enhanced Raman Spectroscopy Measurements

The main database used to train the machine learning models was built from SERS spectra measured from single BA species (please refer to Fig. 4 for analyte structures and SERS spectra). Two substrates per bile acid were immersed in a 100 µM ethanolic solution for 3 h, and then gently dried with a stream of nitrogen gas. Two more substrates immersed in pure ethanol were used as blanks. Four sets of 100 spectra were recorded in distinct areas (each with 10 × 10 spots covering a 40 × 40 µm area) on each substrate using the laser scanning system. An average spot-to-spot variation in SERS signal intensity of 5% was determined for bile acid and blank substrates. A total of 4800 spectra were measured, that is, 800 spectra per bile acid species and 800 spectra from blank substrates.

The performance of the model was tested using an additional dataset (in place of the initial test set) generated with SERS substrates prepared by immersion in solutions contained either TCDCA, DCA, or LCA in varying concentrations (10 µM, 25 µM, 50 µM, and 100 µM) in ethanol. The immersion time and the number of spectra recorded per substrate were reduced to 1 h and 300 spectra, respectively, to increase the analytical throughput. These measurements required several immersion and measurement sequences for each substrate, with thorough rinsing with ethanol between each solution.

### Spectra Processing and Machine Learning

Note: The procedures presented in this section were performed using a collection of tools (i.e., CNN, data enhancement, and spectra preprocessing) written in Python that are freely available on the GitHub repository.^26^ The package also includes a user guide, the CNN model, and the spectral dataset used for model training.

#### Preparation of the Spectral Database

The measured SERS spectra were stored in a single database, with spectra sorted by line, and Raman shifts sorted by column. Labels were assigned in this database to indicate which bile acid species corresponds to which spectrum. These labels were one-hot encoded, meaning that they were stored as a vector with several binary fields associated with a given bile acid, with “1” indicating the presence and “0” the absence of the corresponding species. The database was randomly separated into three new sets with distinct functions: a training set to train the model, a validation set to optimize hyperparameter selection (i.e., user-defined model parameters) and validate the model architecture, and a test set to evaluate the model’s performance. The training set contains 70% of the original database spectra, while the validation and test sets each contain 15%. Once separated, these sets of spectra were kept apart for the rest of the procedure to avoid introducing any form of bias.

#### Data Augmentation

Generally, the more data is used to train a deep learning model, the better its performance, and especially its generalizability, when used on new data. Insufficient data also increases the risk of overfitting and, hence, of learning irrelevant features or patterns such as sampling errors or noise. However, collecting a large number of SERS spectra can be a tedious process given the experimental conditions and substrates that change over time. To address this, the size of the training set was increased using data augmentation, a technique that generates new data by introducing variations to existing data.^27,28^ Since it requires only a few lines of programming, this procedure is simpler and faster than collecting new spectra and is worth considering for any application combining SERS or other spectroscopy with deep learning. The spectra were treated numerically to produce a large and varied training set. Noisier spectra were generated by randomly adding gaussian noise. Spectra were subjected to randomly selected multiplicative factors, intensity offsets, linear slopes, and random vertical shifts to introduce signal background variations. Finally, the number of spectra was increased using a linear combination method inspired by the so-called “Mixup” method used in image classification to produces spectra that not only feed the database, but also regularize and calibrate the models in order to reach better predictions when dealing with SERS spectra coming from multicomponent mixtures.^29,30^ In short, this method creates new spectra by summing two or three spectra belonging to the same or different classes, each multiplied by a factor varying between 0 and 1 that summed together gives 1. These factors were simultaneously applied to the labels, to regularize the model predictions according to the spectrum mixture, resulting in soft labels (e.g., [0.3, 0.1, and 0.6]) instead of the previous hard, hot-coded labels (e.g., [0, 0, and 1]).

#### Spectrum Preprocessing

Although CNN models tend to perform well without data preprocessing, SERS spectra of bile acids were preprocessed using methods widely used in vibrational spectroscopy chemometric analysis to improve their interpretability and to facilitate the introspection of the CNN model.^31,32^ First, a median filter with a size of three values was used to remove spurious signals due to cosmic rays. The spectra were smoothed using a Savitzky–Golay seven-point filter with order 0 and degree 3. Following this smoothing step, the background signals, which are mostly caused by the coverslip glass and surface impurities, were subtracted using a baseline correction method ALS.^33^ Finally, the spectra were normalized using the Euclidean standard, which is calculated for a spectrum as the square root of the sum of all its squared pixel values.

#### CNN Model Architecture

**Figure 2.**
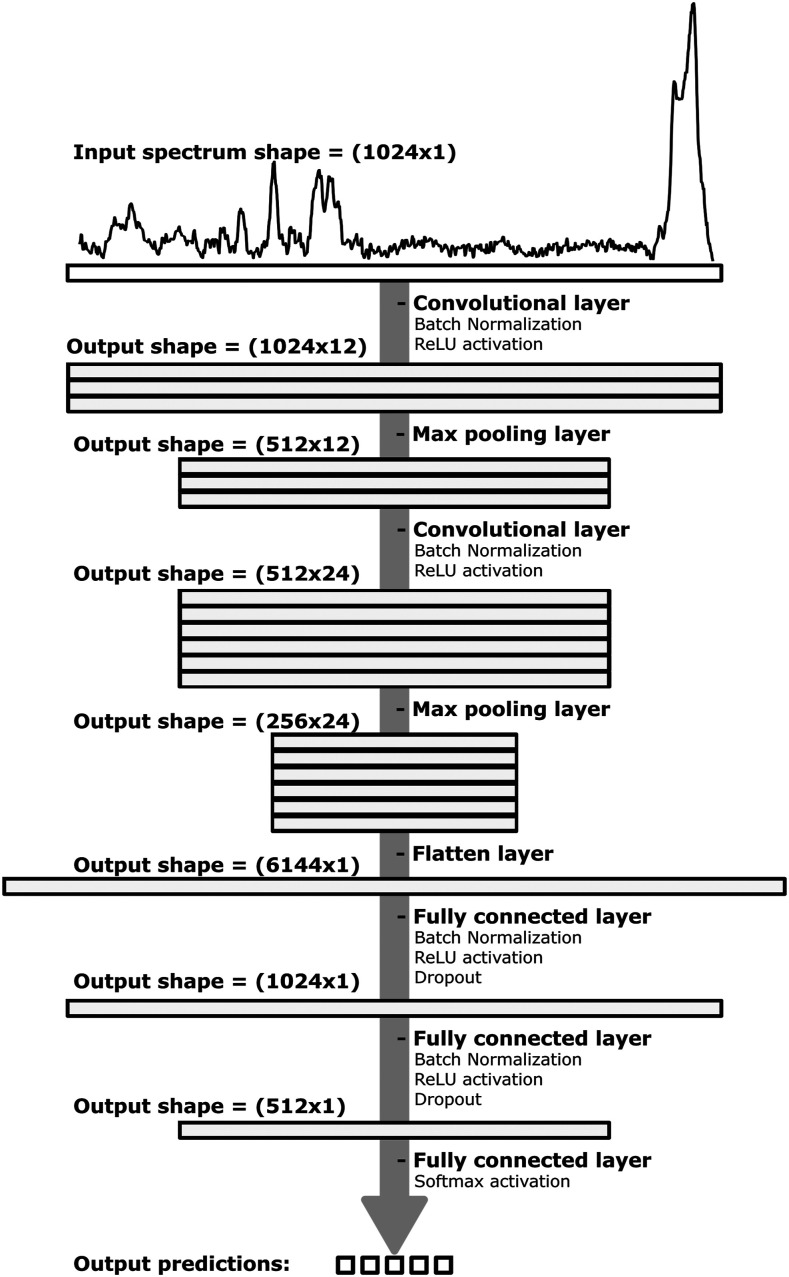
Representation of the architecture of the convolutional neural network (CNN) model. From top to bottom, the model includes two convolutional layers, 12- and 24-kernel filters (filter size = 5 × 1), each followed by a max-pooling layer, and two fully connected layers of 1024 and 512 nodes.

The model, shown in Fig. 2, was built using the Keras library with TensorFlow backend support.^34^ From spectrum input to output prediction, this model is mainly composed of two convolutional layers of 12 and 24 kernel filters (filter size = 5 × 1) and two fully connected layers of 1024 and 512 nodes. Each convolutional layer is followed by a maxpooling layer (filter size = 2 × 1) that halves the principal dimension of the output data. A flattened layer of 6144 units connects the output of the maxpooling layers to the first fully connected layer. With the exception of the output layer, a rectified linear unit (ReLU) activation function is used for convolutional and fully connected layers. The Softmax activation function is used for multiclass classification at the model’s output. To minimize overfitting and speed up convergence during model training, batch normalization is applied before each ReLU activation function and dropout (rate = 0.45) after the two dense layers.^35–37^ Model training was performed on a single Nvidia Quadro P600 GPU using an Adam optimizer (learning rate = 0.001) with a batch size of 132 spectra and a cross-entropy loss function.^38^ Training duration was restricted to a maximum of 50 epochs with a programmed early stop to interrupt the training when the model no longer improves. To monitor the model’s performance during the learning process, both loss and accuracy were evaluated after each epoch on the training and validation sets to ensure that it generalized well to the new spectra and did not overfit during the process (Fig. S3, Supplemental Material, for examples of loss and accuracy learning curves obtained during CNN training). For comparison purposes, a PCA-LDA model based on 10 principal components (∼95% of the cumulative covariance) was also developed using the basic frameworks provided by the Python Scikit-Learn library.

## Result and Discussion

### Gold Nanostars (AuNSt) and Surface-Enhanced Raman Spectroscopy Substrate Characterization

**Figure 3.**
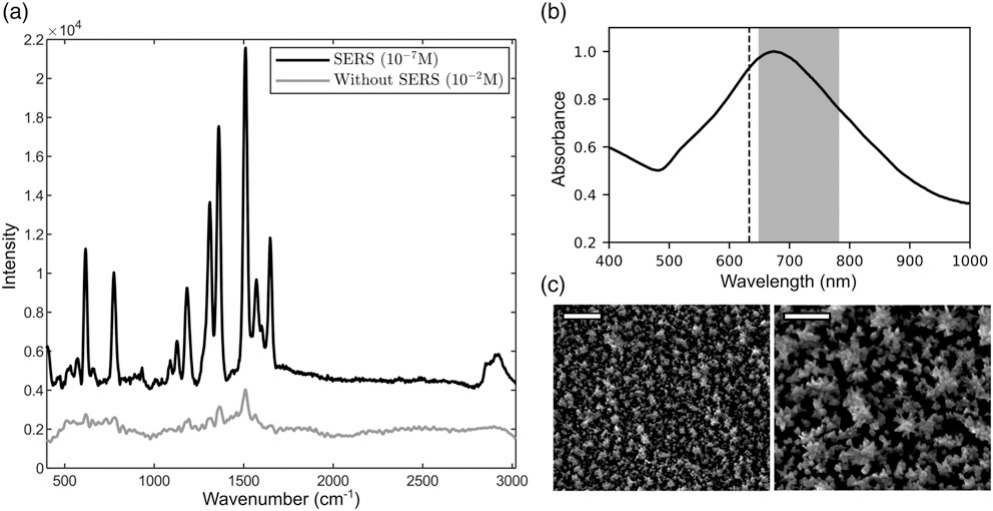
(a) Comparison between surface-enhanced Raman spectroscopy and Raman spectrum of Rhodamine 6G at different concentration, integration time = 5 s. (b) UV–Vis absorption spectrum of AuNSt SERS substrates. Dashed line and gray area correspond respectively to the excitation wavelength of 632.8 nm and Raman’s band scattering region. (c) Scanning electron microscopy (SEM) images of AuNSt SERS substrates; Scale bars = 1 µm (left), 200 nm (right).

As a plasmonic material, gold was preferred to silver as it is more chemically stable for in vivo measurements.^39^ In addition, gold nanoparticles can be excited using red or near infrared wavelengths that are less prone to cause cell phototoxicity or auto-fluorescence.^40^ Star-shaped nanoparticles (Fig. 3) were chosen as plasmonic enhancers to take advantage of the high electric field regions at the ends of the tips and the substantial signal enhancement in SERS.^41^ Sizable SERS enhancement is expected from these AuNSt since their absorbance band (Fig. 3b) is centered at 706 nm and covers both the excitation wavelength of 632.8 nm and the measured Raman region from 650 to 780 nm.^2^ SEM images of the substrates (Fig. 3c) show that the well-defined tips of the gold nanostars (AuNSt) is conserved after immobilization on glass coverslips previously functionalized with DMAET and OCT. An average surface density of (470 ± 20) AuNSt/µm^2^ was measured from three substrates and three distinct regions used for each substrate (Fig. S2, Supplemental Material), a value comparable or higher than values reported in the literature for this type of nanoparticles.^42–44^ An RSD of 4.3% suggests a suitably uniform and repeatable surface coverage of the SERS substrates. Rhodamine 6G (R6G) was used to evaluate the Raman signal enhancement provided by the substrates, as it is commonly used for this purpose.^45,46^ The R6G SERS and Raman spectra presented in Fig. 3a) highlight the enhancement provided by SERS. An average apparent enhancement factor of $1.1\times 10^{6}$ was determined using the bands centered at 1335 and 1523 cm^−1^ (see the Supplemental Material for details on calculating this value).

### Features of the CNN Model

Given the weak SERS spectral signatures and the close similarity between the molecular structures of bile acids, a high-performance model able to capture slight spectral variations was required to identify and classify the SERS spectra of these molecules. CNNs are powerful deep neural networks that are widely used for various tasks such as image classification and speech recognition^47–51^ and that are also seeing increasing use for the analysis and classification of spectra. Indeed, previous studies have reported that CNN models achieve better results than other classification methods for classifying Raman, Fourier transform infrared, and SERS vibrational spectra. For instance, in SERS, a CNN model was recently used to measure in vitro the gradients of eight metabolites near different cell lines.^52^ In another study, a CNN model was applied to distinguish 20 commonly used pesticides.^16^ In the present contribution, we are specifically targeting the development and application of a CNN model to the classification of very similar molecular structures, as well as proposing more elaborate data augmentation and fine-tuning methods. Before presenting the performance of our model, a brief overview of the features and capabilities of neural networks and CNNs pertaining to spectral classification is provided in this section.

Neural networks are made up of layers, the highest-level building blocks in deep learning. Each layer is composed of computing units (“neurons”) connected through one or more weighted connections to the neurons of a previous or subsequent layer. The basic framework of a neural network includes an input layer, one or more intermediate layers called hidden layers, and an output layer. As data is propagated through the model, each neuron receives input data, transforms it using an activation function, and transfers the resulting data to the neurons of the subsequent layer that will perform the same process. Training a model essentially consists of fitting the weights applied to the connections between the neurons of different layers, and the process can be summarized in two steps repeated several times. In the forward propagation step, the parameters of the input samples are propagated and transformed through the network to reach the output layer and give the predicted output values. In the backpropagation step, an error is calculated by comparing the predicted values with the expected values and propagated backwards through the neural network to update the weights in the neural network.^47^

Convolution and pooling operations are two key elements specific to CNN that preprocess incoming data prior to classification and allow CNN models to outperform traditional neural networks in most cases. These two operations improve the quality of the data before propagating it to the neural network itself. The pooling layer divides the total range of input data into several sub-regions of equal size, filters them one by one, and returns a unique value for each of these sub-regions, thereby reducing the spatial dimensions of the data. The maxpooling layers used in our model return each subregion’s maximum value as output. The convolutional layers apply convolution filters, also known as kernel filters, to the incoming data. These filters are one-, two-, or three-dimensional matrices that are widely used in image processing for edge detection, sharpening, and other image processing tasks. The values governing the filters of the convolution layers are updated during model training and unlike traditional dense layers where the updated weights consider individual input values separately, convolutional updated filters are instead applied to several adjacent input values at the same time, allowing efficient capture and extraction of the spatial components that are most relevant for a desired task. With several distinct filters applied within a single convolutional layer, this process also enriches the information contained in the transferred data. Furthermore, since vibrational spectra are vectors with spatially dependent units, the use of convolutional layers is fully justified, and the trained filters are expected to be able to detect band edges and reduce noise or signal background.^53,54^ By combining convolutional layers with pooling layers, the first part of the model is then able to produce new data that are both reduced in size and information-enriched before forwarding them to the second part of the model for classification. This first part also gives a considerable advantage to CNN models by integrating a large part of the data preprocessing, which significantly reduces the work required in data preprocessing.

### CNN Model Testing on Surface-Enhanced Raman Spectroscopy spectra of Bile Acids

**Figure 4.**
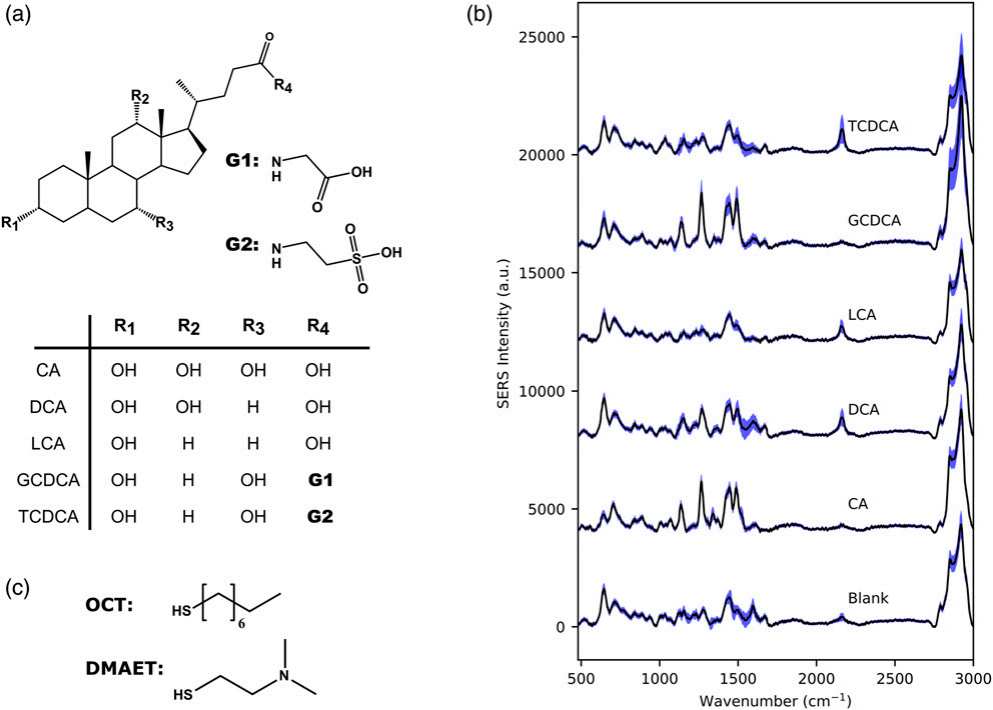
(a) Molecular structures of bile acids used in this work: Primary acid (cholic acid, CA), two secondary, unconjugated acids (deoxycholic acid, DCA and lithocholic acid, LCA), and two conjugated forms (glycochenodeoxycholic acid, GCDCA, and taurochenodeoxycholic acid, TCDCA). (b) Averaged surface-enhanced Raman spectroscopy spectra (*n* = 600) for each bile acid (standard deviation indicated by light blue areas). Concentration was 100 μM with a 2.5 s integration time. (c) Averaged surface-enhanced Raman spectroscopy spectrum from a blank substrate is also presented and shows Raman bands associated with ligands OCT and DMAET. A baseline correction was performed on all spectra using the asymmetric least squares method.

To build the dataset for the CNN model, the five bile acids were measured at concentrations of 100 µM (Fig. 4). This is the first reported example of using SERS to detect members of this family of closely related chemical structures. Based on conventional Raman spectroscopy measurements performed on pure BA samples, we were able to identify several bands in the SERS spectra of the bile acids. The band around 1020–1050 cm^−1^, slightly more intense for TCDCA, was assigned to the symmetric S=O deformation of the sulfonate group. More pronounced bands were observed for GCDCA, DCA, and CA around 1300 cm^−1^ and were associated with the C–O bending of carboxylate and carboxylic acid groups. The region between 1400 and 1700 cm^−1^ presents several partially unresolved bands that may be associated with C=O stretching modes (∼1400 and 1640 cm^−1^) as well as CNH and CH_2_/CH_3_ deformations (∼1540 cm^−1^ and ∼1445 cm^−1^, respectively). Lastly, the region between 2860 and 2930 cm^−1^ associated with OH and CH_2_/CH_3_ stretching bands presents some differences between the various bile acids. Many of the remaining bands in the SERS spectra could not be assigned, possibly due to interference from the functionalization layer.

Bile acid spectra are significantly less intense than that of R6G measured using the same substrates, which was expected given the low Raman cross-sections reported for similar steroid-like molecules.^55^ Furthermore, since they share several functional groups with Raman bands in the same locations, the challenge posed by the identification of one species from another is an excellent opportunity to evaluate the performance of the CNN model. In particular, the spectra measured for bile acid species CA and GCDCA and the blank substrate were exceedingly similar.

**Figure 5.**
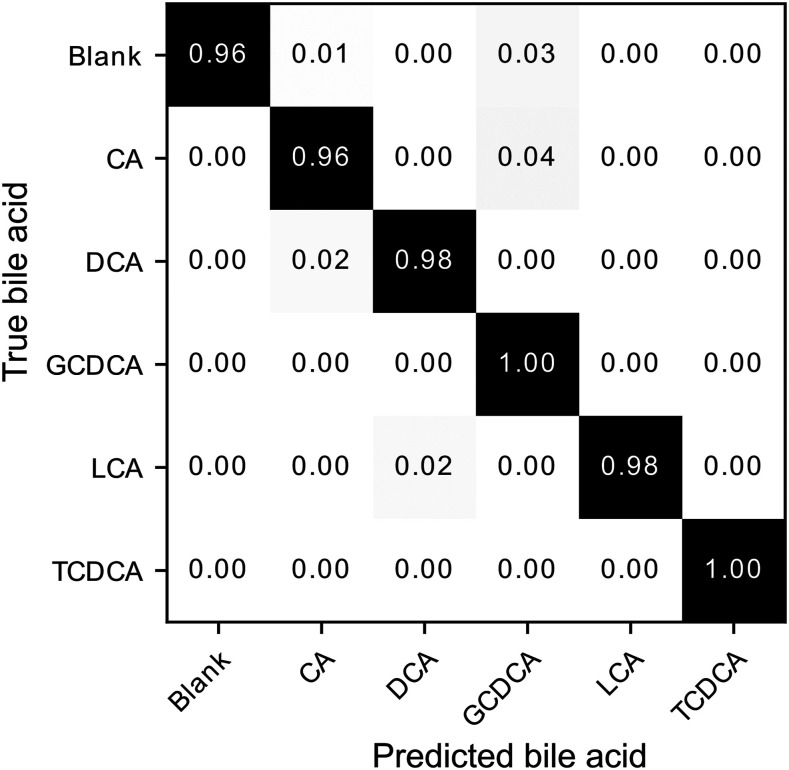
Row-normalized confusion matrix computed using the CNN model predictions on the test set comprising 720 surface-enhanced Raman spectroscopy bile acid spectra. Prior to row normalization, the values contained in the confusion matrix were averaged over the predictions resulting from 10 independently trained model versions.

A confusion matrix was used to evaluate the efficiency of the CNN model for SERS measurements of the five bile acid species considered in this work (Fig. 5). It summarizes and sorts the model’s predictions according to the true expected bile acid (sorted by columns), the bile acid predicted by the model (sorted by rows), and reveals which bile acid species the CNN model confounds with which other, and to what degree. The results show that the model managed to identify and differentiate the five bile acid species despite their close similarity and the absence of distinct features in their SERS spectra. The correct predictions made by the model are found on the diagonal cells and account for a large majority of all classifications, with a rather small proportion of the spectra leading to erroneous predictions. Overall, the CNN model classified the spectra with an accuracy of (98.1 ± 0.6) % over 9 independent runs (Table S1, Supplemental Material), while at most 90.2% were correctly classified using the PCA-LDA method (with 10 principal components) commonly used in Raman spectroscopy, thus showing that the model repeatedly succeeds in correctly classifying bile acids. These results confirm the capabilities of the CNN model to discern small variations in nearly identical spectra to differentiate and classify species with very similar molecular structures. Interestingly, it can be postulated that the central, essentially featureless region between 1750 and 2750 cm^−1^ is wasted on the spectrometer, and that the model could perform as well or better without it. To validate this hypothesis, we retrained and tested the CNN on the same spectra used in the paper, firstly bounded at 1750 cm^−1^ to the right to keep only the fingerprint region, and again with the central part (1750–2750 cm^−1^) removed, in effect stitching together the fingerprint and OH–CH regions (Fig. S4, Supplemental Material document for the confusion matrices). We found that numerically removing the central region of the spectra does not, as expected, impact the accuracy of the model (97.0 ± 0.8% versus 98.1 ± 0.6%), while reducing the computation time needed to train the CNN model. On the other hand, using only the fingerprint region significantly reduces the accuracy (90.0 ± 0.1%) and it becomes more difficult to separate the bile acids. It can therefore be hypothesized that using a higher spectral resolution, for example, by moving from a 1180 lines/mm grating (spectral resolution ≅ 0.22 nm) to a 2400 lines/mm grating (spectral resolution ≅ 0.11 nm) and stitching together the fingerprint and OH–CH regions, might result in improving further the accuracy of the CNN model.

The confusion matrix also shows that the two most problematic bile acids are GCDCA and CA. For the GCDCA, the CNN model correctly classified all its true spectra, but erroneously assigned 4% and 3% that should have been assigned to CA and the blank substrate. These results suggest that the predictions of the CNN model are less accurate for GCDCA than for the other bile acids. For CA, the model correctly classified 96% of its true spectra and erroneously assigned 2% and 1% that should have been assigned to DCA and the blank substrate. The CNN model predictions assigned to CA are more accurate than those of the GCDCA, but the model is less sensitive and fails to correctly detect all of the true CA spectra.

**Figure 6.**
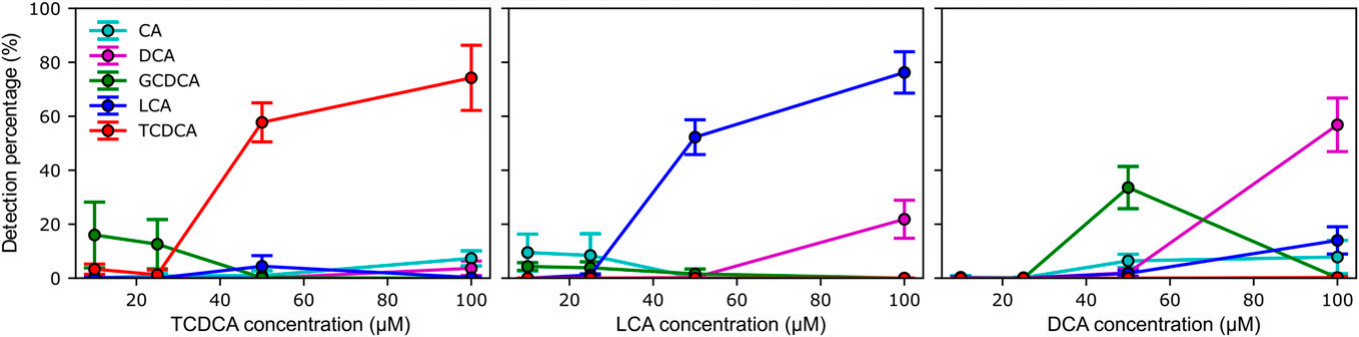
Percentages detected for each bile acid following model predictions performed on surface-enhanced Raman spectroscopy measurements of single-species solutions of TCDCA, LCA, and DCA at different concentrations (10, 25, 50, and 100 µM). A total of 300 spectra were measured for each concentration. The percentages are averages over the results obtained with 10 independent versions of the trained model and the error bars correspond to the calculated standard deviation.

Finally, the CNN model was also tested with solutions of single species at lower concentration. Due to their higher classification score (Fig. 5), TCDCA, LCA, and DCA bile acids were used for SERS measurements at different concentrations. It should be pointed out that whereas TCDCA is structurally distinguishable by its amide and sulfonate groups, the molecular structure of DCA and LCA is almost identical, differing by a single hydroxyl group. To this end, the model was fine-tuned to improve its predictive performance with respect to experimental variations between the spectra from the training set and the spectra from this experiment. This procedure consisted in re-training the pretrained CNN model with an additional training set consisting of new spectra measured on blank substrates and a random set of 30% of the spectra used for the previous training. To preserve most of the CNN model parameters optimized during the first training, the weights of the first layers (convolutional and maxpooling layers) were frozen during the fine-tuning process while the learning rate and number of epochs were reduced to 0.0001 and 5 epochs, respectively. As shown in Fig. 6, the CNN model was again able to correctly classify spectra for the three bile acids at 100 µM despite slightly different experimental conditions such as the use of different SERS substrates. The model also succeeded to detect TCDCA and LCA at 50 µM, but failed to detect DCA, confusing it with GCDCA. This behavior occurred only for the 50 µM DCA solution, suggesting that the predictions are erroneous for this measurement since GCDCA is not detected on the same substrate at lower or higher concentrations. The confusion matrix (Fig. 5) revealed that a higher number of spectra were misclassified into the GCDCA class during model training. Therefore, several spectra that should have been attributed to the blank substrate or DCA bile acid were likely misclassified as GCDCA. Below 50 µM, the CNN model failed to detect all three bile acids in solution. Importantly, the specificity of the CNN model is demonstrated by the small number of false predictions below 50 µm, with occurrences of spectra attributed to bile acids absent from the solution not exceeding 20%.

## Conclusion

This study demonstrates for the first time the detection of bile acids, a family of very similar molecular structures and spectral signatures, using SERS spectroscopy and deep learning. The CNN model developed herein was able to successfully classify the five bile acids under study from their SERS spectra with a high accuracy of (98.1 ± 0.6) percent. Moreover, the model remained robust across different bile acid concentrations, thus validating the potential of the tandem use of SERS spectroscopy and deep learning models. While the concentrations used in this proof-of-concept study may appear supraphysiological, one may consider that intestinal elimination is the main route for bile acid elimination. Indeed, total bile acid levels greater than 2337 µmol/48 h are considered as indicative of bile acid malabsorption^56^ and proposed as a diagnostic marker for IBD by the Mayo clinic.^57^ While it is extremely difficult to extrapolate concentrations from aqueous standards to fecal levels measured over a specific period, one may speculate that the 100 µM used here remains relevant from a pathological point of view. As mentioned above, 95% of bile acids secreted in the duodenum are reabsorbed in the ileum.^6^ Thus, it can be envisioned that in the small intestine, bile acids levels may be even more elevated. And whereas resolving mixtures of bile acids undoubtedly represents an equally important challenge, the model’s performance may be improved further by, for example, increasing the resolution of raw SERS spectra included in the training database, as discussed above. Furthermore, by providing a ready-to-use, freely available, and powerful CNN model,^26^ we hope that this will promote the application of CNN models and other deep learning tools to SERS spectroscopy.

## Supplemental Material

sj-pdf-1-asp-10.1177_00037028221077119 – Supplemental Material for Pushing the Limits of Surface-Enhanced Raman Spectroscopy (SERS) with Deep Learning: Identification of Multiple Species with Closely Related Molecular Structures Supplemental Material, sj-pdf-1-asp-10.1177_00037028221077119 for Pushing the Limits of Surface-Enhanced Raman Spectroscopy (SERS) with Deep Learning: Identification of Multiple Species with Closely Related Molecular Structures by Alexis Lebrun, Hubert Fortin, Nicolas Fontaine, Daniel Fillion, Olivier Barbier, and Denis Boudreau in Applied Spectroscopy
