# Supplementary material for: Pushing the Limits of Surface-Enhanced Raman Spectroscopy (SERS) with Deep Learning: Identification of Multiple Species with Closely Related Molecular Structures
Source: Appl Spectrosc. 2022 Mar 26;76(5):609–19. doi: 10.1177/00037028221077119 (PMC9082968; doi:10.1177/00037028221077119)
Supplement: sj-pdf-1-asp-10.1177_00037028221077119 – Supplemental Material for Pushing the Limits of Surface-Enhanced Raman Spectroscopy (SERS) with Deep Learning: Identification of Multiple Species with Closely Related Molecular Structures [file sj-pdf-1-asp-10.1177_00037028221077119.pdf]

Pushing the Limits of SERS Spectroscopy with Deep Learning: Identification of multiple Species with closely related molecular structures.

Alexis Lebrun[a,b], Hubert Fortin[a], Nicolas Fontaine[a], Daniel Fillion[a], Olivier Barbier[b] and Denis Boudreau[a]

[a]Departement of chemistry, Université Laval, Québec, QC, Canada

[b]Laboratoire de pharmacologie moléculaire, Axe Endocrinologie-Nephrologie, Centre de recherche du CHU de Québec, Université Laval, QC, Canada

Materials and Methods

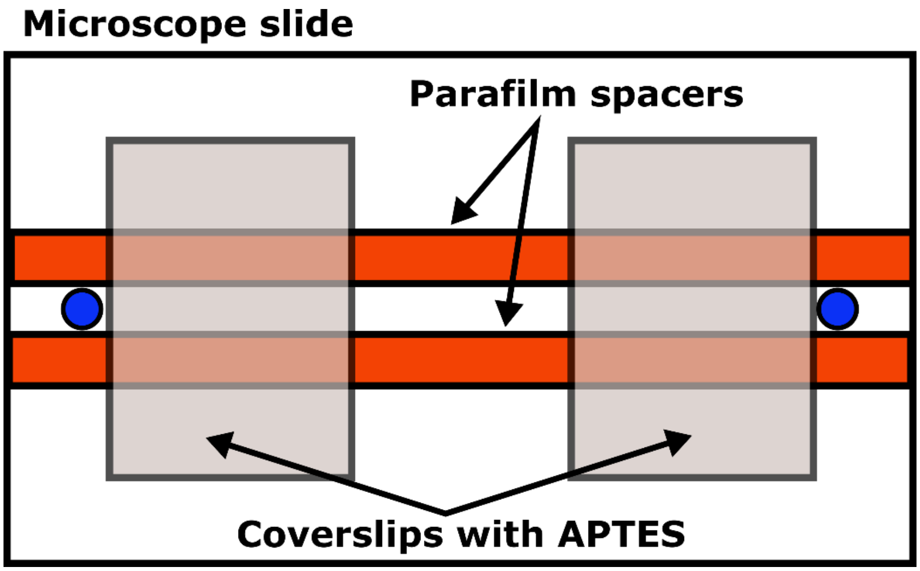

Figure S1: Schematic representing two fluidic chambers used to immobilize gold nanostars (AuNSt) on microscope coverslips previously functionalized with (3-Aminopropyl) triethoxysilane (APTES). Two pieces of Parafilm are used as a spacer between the APTES coverslips and a microscope slide which acts as a support, and delimits the area where the AuNSt solution will be introduced. The blue dots indicate the inlet points used to introduce the AuNSt solution into the fluidic chambers

Results and discussion

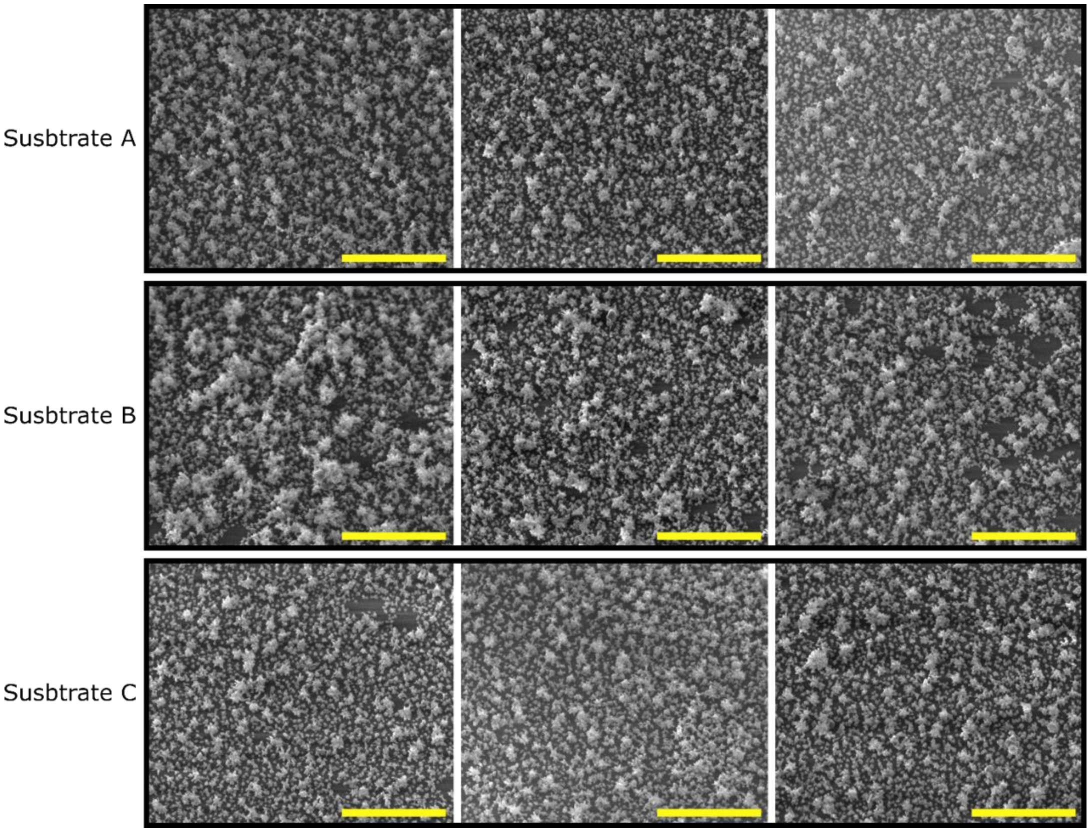

Figure S2: Raw SEM images acquired at random locations on developed SERS substrates. Three images were acquired per substrate for a substrate triplicate (A, B, C), giving a total of 9 images. Scale bar = 1 µm.

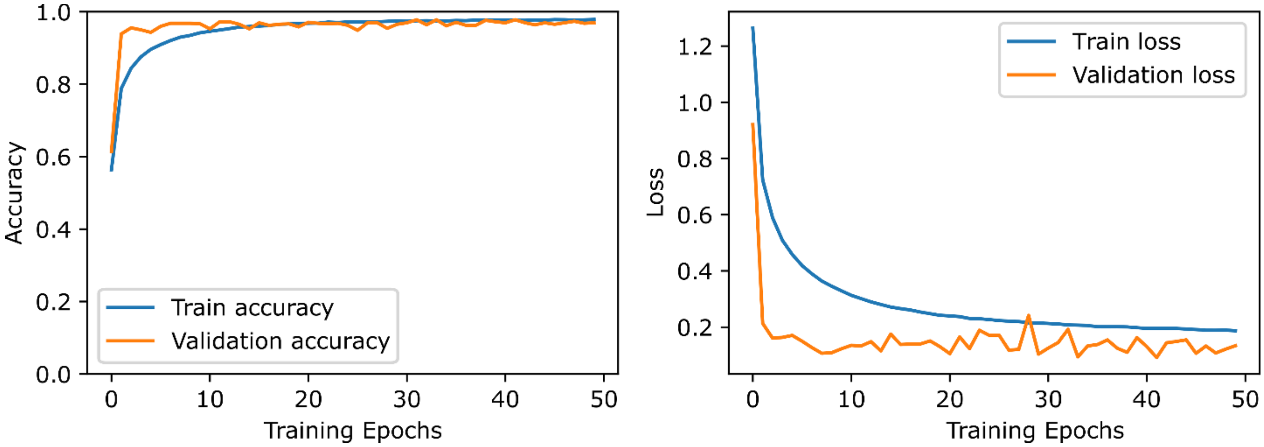

Figure S3: Accuracy and loss learning curves obtained for training and validation sets during a CNN training session. The data shows no sign of overfitting after 50 epochs.

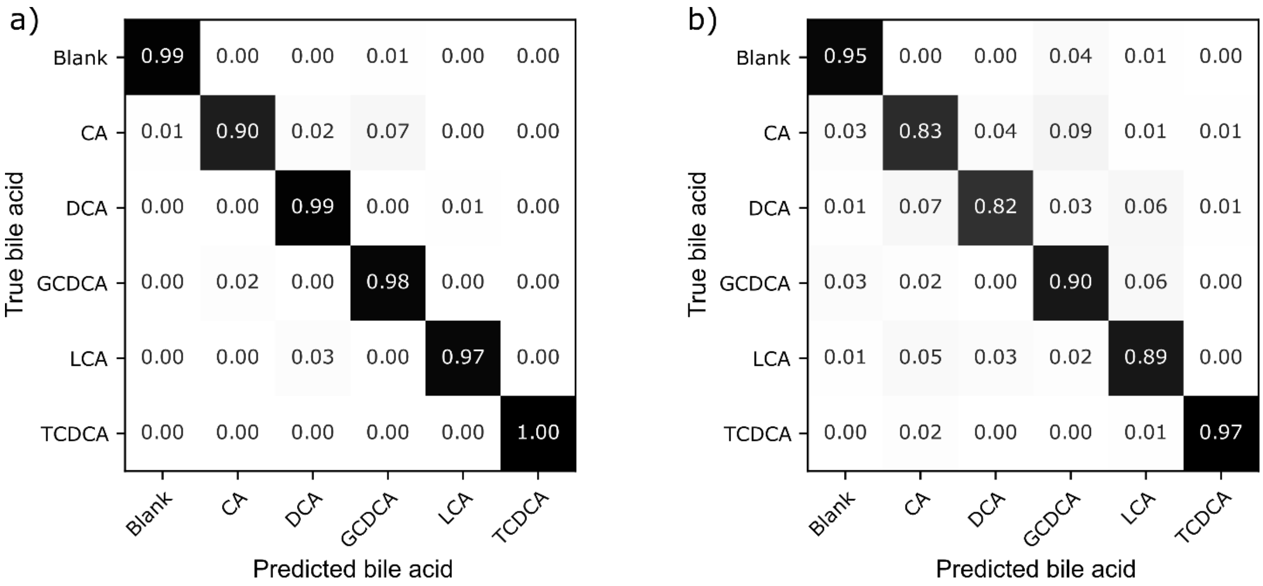

Figure S4. Row-normalized confusion matrices computed using CNN model predictions on the test set comprising 720 SERS bile acid spectra with (a) the center region of the spectra (between 1750 and 2750 cm^{-1}) removed and (b) only the left part (value below 1750 cm^{-1}) retained. Prior to row normalization, the values contained in the confusion matrixes were averaged over the predictions resulting from 10 independently trained model versions.

**Independent run**	**Validation accuracy (%)**
**1**	98.5
**2**	99.0
**3**	98.0
**4**	97.3
**5**	97.8
**6**	97.5
**7**	97.5
**8**	98.3
**9**	99.2
	**98.1±0.6**

Table T1: Accuracy values calculated on different validation sets (N=720 spectra) for 9 independent runs.

Determination of apparent SERS enhancement factor

Nanostar-coated and functionalized SERS substrates were prepared as described in the main text and used for SERS signal acquisition. Blank glass slides were cleaned in a Pirahna solution (1:1:1 $H_2SO_4/H_2O/H_2O_2$), rinsed in water and ethanol and used as is for standard Raman signal acquisition, i.e., without silanization. Both substrates were immersed in Rh6G solutions for 3 hours, rinsed with water to removed physisorbed Rh6G molecules, gently dried with nitrogen and measured with the confocal Raman microscope. Due to the lower sensitivity of Raman compared to SERS, a higher concentration of Rh6G was used for standard Raman than for SERS (10 mM vs. 0.1 µM). Laser power and integration time ((2.8 ± 0.3) mW and 5 sec, respectively) were the same for both substrates. The signal was measured at 1335 and 1523 cm^{-1} and an average EF was computed.

We used the following formula to calculate the EF:

$$EF = \frac{I_{SERS}}{C_{SERS}} \frac{C_{Raman}}{I_{Raman}}$$

where I_{SERS} is the measured SERS signal intensity, I_{Raman} is the measured Raman signal intensity, C_{SERS} is the concentration of the Rh6G solution used for SERS measurements and C_{Raman} is the concentration of the Rh6G solution used for the standard Raman measurement. This expression differs from the one recommended by Bell et al.[1] in the use of N_{SERS} and N_{Raman}, where N is the number of molecules being excited. Obviously, our calculation method suffers from several approximations, due in great part to the different nature of the substrates (nanostar-coated glass slide for SERS, bare glass surface for Raman) and the uneven distribution of analyte molecules due to the drying process. Moreover, using different substrates with slightly different thicknesses also means that we are unsure of the beam footprint and, hence, the power density at the surface. It is important to point out that the EF factor determined herein should be used as a qualitative indication of the signal enhancement provided by the nanostar-coated substrates and not for comparing with other works in the literature.

[1] Bell SEJ, Charron G, Cortés E, Kneipp J, Lamy de la Chapelle M, Langer J, Procházka M, Tran V, Schlücker S. Towards Reliable and Quantitative Surface-Enhanced Raman Scattering (SERS): From Key Parameters to Good Analytical Practice. Angewandte Chem. Int. Ed. 2019 Oct; 59(14): 5454-5462.
